# Boston Society for Medical Improvement

**Published:** 1881-06

**Authors:** 


### Reports of Societies.

### PROCEEDINGS OF THE BOSTON SOCIETY FOR MEDICAL IMPROVEMENT.

*Chronic Endarteritis of the Aorta, With Unusual Course and Consequences.*—Dr. Draper exhibited the aortic arch from the body of a woman forty-five years old, and presented brief notes of the clinical history of the case. Symptoms referable to the disease represented by the specimen began to develop in 1875, when the patient first had severe shooting pains in the left side of the neck, and between her shoulders; these were attended with dyspnœa, sometimes amounting to orthopnœa, symptoms which persisted during her life. At intervals of two or three months, between 1875 and 1879, she had attacks, which she described as "spells coming without warning, seeming as if a wave struck her on the back, rolled up over her shoulder, and broke as it crossed her head." During these nervous paroxysms her urine became dark-colored, and micturition was painful; the attacks ended in insensibility, the patient falling wherever she might happen to be.

In 1876, her attending physician, Dr. W. H. Campbell, of Roxbury, observed a slight fullness above and below the sternal end of the left clavicle; he detected some pulsation at this point, and a sound which he interpreted as an aneurisnal bruit. At this time, also, there was a very perceptible difference between the two radial arteries, the impulse in the right being normal, that in the left being soft and small. Nothing wrong was observed about the heart.

In August, 1879, the patient was found, one evening in an unconscious condition on the street, and was thereupon admitted to the City Hospital, where she was under Dr. Draper's observation for six weeks. At this time the prominence over the clavicle was not apparent. The left radial pulse was relatively much weaker than the right, and at times was wholly obliterated. The left carotid pulse was also scarcely perceptible. The voice was tremulous, and at times husky. The pupils were equal, although the patient stated that she had noticed a dilation of the left pupil coincidently with the attacks of pain and dyspnœa. A musical murmur was heard above the middle of the left clavicle over a limited area. Nothing wrong was noticed about the heart. Under treatment the patient grew stronger, and her general condition improved; the œdema of the feet and ankles, somewhat annoying at the time of admission, subsided; the souffle above the clavicle was less distinct. Pain in the left arm developed during the latter part of her stay, but there was no change in the appearance of her arm. While a patient at the hospital she had no recurrence of the neurotic attack.

During the eighteen months after her discharge from the hospital. and up to a few days before her death, she was able to live in comparative comfort most of the time.

An occasional exascerbation of her previous symptoms occurred. Dr. Campbell noticed in the autumn of 1879 that the musical sound had wholly disappeared from above the clavicle, and the left radial pulse was lost. The pain in the arm continued. A fortnight before her death she had an attack simulating angina pectoris. She was found dead in her bed one morning, having retired in usual health the night previous.

At the time, the chief interest was found to centre in the heart and arch of the aorta. The heart was hypertrophied to a marked degree, and there was a moderate amount of dilatation of the left ventricle. The muscular tissue was of good color and firmness. All the valves were normal except the aortic, whose semi-lunar folds were thickened, retracted, and irregular, constituting a stenosis as well as incompetence of the aortic opening. The coronary arteries were healthy. The aorta throughout its extent was thickened, roughened, and reduced in caliber. Thin plates of calcareous matter were imbedded at intervals in the intima. But the most interesting feature was the fact that in the process of inflammation and consequent alteration in the coats of the artery, the orifice of the left common carotid had become wholly occluded by a semi transparent tissue thrown across it, and presenting on the inner (aortic) side a small funnel-shaped depression, while the orifice of the left subclavian was in a similar manner almost obliterated, the opening for the passage of blood being reduced to a mere point, admitting a fine probe. Both the arteries (carotid and subclavian) above this obstruction were of normal size, and their walls were, apparently, a little thinner than usual. The innominate, on the other hand, was dilated so as to admit the tip of the index finger to the distance of nearly an inch; its intima in this dilated portion shared in the inflammatory process of the aorta. Above this part the innominate was normal. Other organs and regions of the body presented nothing noteworthy so far as they were examined. The head was not opened.

*Hirsuties.*—Dr. White made some remarks on the use of electrolysis in the treatment of hirsuties, and exhibited a battery adopted for this purpose.

*Lithotrity.*—Dr. Francis Williams, by invitation, described and exhibited a new evacuator for the bladder, the report of which will appear in a later number of the Journal.

Dr. Bolles thought that the valve, which seemed to him similar in principle to that of the "flute-key" valve of a stomach pump, might be used with advantage in connection with a siphon for filling, emptying or washing the stomach without the usual stomach pump syringe. Its compactness and simplicity would commend it for that purpose, and the readiness with which the current can be reversed makes it more desirable than the siphon alone.—*Boston Medical aad Surgical Journal.*

